# Gaining the Upper Hand? Further Evidence of Pain as a Pleasurable Experience and the Unexpected Relationship Between Sadomasochistic Sexual Preference and Chronic Pain

**DOI:** 10.1002/ejp.70230

**Published:** 2026-02-07

**Authors:** Annabel Vetterlein, Sarah Kirrinnis, Merlin Monzel, Ana‐Laia König Guasch, Martin Reuter

**Affiliations:** ^1^ Department of Psychology, Personality & Biological Psychology University of Bonn Bonn Germany

**Keywords:** chronic pain, pain and pleasure, sadomasochistic sexual preference

## Abstract

**Background:**

A growing body of research reports positive consequences of acute pain, including emotional self‐regulation. Pain as a pleasurable experience has also been regarded in the context of sadomasochistic (SM) interest, albeit quantitative empirical evidence is scarce. Recently, an elevated prevalence of chronic pain (CP) has been reported in SM practitioners; however, in absence of a control group. To contribute to research in the field, we strove to see whether we could replicate the finding and further aimed to identify psychological predictors of SM.

**Method:**

A total of 617 participants (*N* = 242 with SM sexual preference) completed an online questionnaire battery comprising psychometric instruments for the measurement of pain attitudes, sensation seeking and pain sensitivity. An age‐ and sex‐matched sample was created to control for their respective influence.

**Results:**

CP prevalence in the SM subsample was 47.2%, which we found to be significantly increased compared to the prevalence in the non‐SM subsample (29.4%). Neither sex nor age seemed to explain the relationship. There were no interaction effects of SM × CP on pain attitudes. A hierarchical logistic regression model explained around 42% of the variance in SM, with CP, sensation seeking, and viewing pain as a challenge as significant predictors.

**Conclusions:**

We replicated an increased CP prevalence in SM and ruled out previous sampling biases. We further extended evidence on factors predicting SM sexual preference. Large‐scale, representative and prospective studies are needed to corroborate the idea of SM as a coping strategy used by some CP patients.

**Significance Statement:**

We demonstrated a significantly elevated chronic pain prevalence of around 47% in individuals with sadomasochistic sexual preference, ruling out previously discussed sampling biases. Possibly, sadomasochistic practise is used as a coping strategy by some chronic pain patients. We further presented a model of pain‐related and psychological variables explaining around 42% of variance in sadomasochistic sexual preference in general. The results are discussed in terms of their potential to inform pain management strategies.

## 1 Introduction

Historically, the majority of pain research has regarded pain in light of its aversive nature. Only a few researchers have looked at ways in which acute pain can be associated with positive consequences (e.g., Bastian, Jetten, and Hornsey 2014; Bastian, Jetten, Hornsey, and Leknes 2014; Dunkley et al. 2020; Leknes and Tracey 2008), although the understanding might hold potential to inform chronic pain (CP) prevention and management. The overarching aim of the present study was to contribute to this sparse body of research. As sadomasochistic (SM) sexual preference can serve as an example of a close pain‐pleasure relationship, we sought to gain further insight into its correlates.

In their review, Bastian, Jetten, Hornsey, and Leknes (2014) compile evidence demonstrating benefits that may arise through the experience of acute pain. According to the authors, pain can facilitate pleasure by providing a contrast relatively enhancing the pleasantness of subsequent stimuli and by amplifying sensory experiences. In a study using a modified cold pressure task, participants who had just removed their hand from ice water after an emergence of 90s reported enjoying a chocolate biscuit significantly more compared to those who had just removed their hand from water at room temperature. In a related study, subjects who had just experienced pain were significantly more sensitive to various types of flavours (Bastian, Jetten, and Hornsey 2014). Moreover, pain can serve as a justification to indulge in pleasures otherwise associated with guilt (Bastian, Jetten, Hornsey, and Leknes 2014). In a study using a social pain paradigm, participants who were told that they were the type of person who would remain without close relationships in the future chose a candy bar over a healthier snack significantly more often than those who were not (Twenge et al. 2002). In addition, Bastian, Jetten, Hornsey, and Leknes (2014) argue that pain offers a means for self‐regulation by inducing mindfulness, by reducing high‐level self‐awareness, and by enhancing cognitive and affective control. For instance, previous research in non‐suicidal self‐injury and healthy samples showed increased emotion regulation during acute pain and at pain offset (Franklin et al. 2010, 2013). Lastly, Bastian, Jetten, Hornsey, and Leknes (2014) consider pain as a means to demonstrate personal virtues and to foster social affiliation. There is, for example, empirical evidence for an enhanced sense of belonging through a shared experience of pain (Gerard and Mathewson 1966; Xygalatas et al. 2013).

The mechanisms through which pain is thought to become a pleasurable experience in SM sexual preference overlap with this, particularly with respect to emotion regulation through mindfulness. Baumeister (1988) put forward the idea that masochism may serve as a coping mechanism replacing high‐level self‐awareness by low‐level awareness, thereby shifting the focus towards bodily sensations in the present moment. More recently, Dunkley et al. (2020) have proposed an integrative model suggesting that an interplay of factors such as learning experiences, context, and perception of control leads to a positive anticipation of pain and sexual arousal, marked by a release of dopamine and oxytocin. Together with further elevated levels of dopamine, cortisol, opioids and endocannabinoids in response to a noxious stimulus (for a review see Wuyts and Morrens 2022), this is assumed to create an altered state of consciousness, mindfulness and eventually, the perception of pain as pleasurable.

Given the previously discussed (for a review see Leknes and Tracey 2008) role of the dopaminergic and opioid system in both pain (see, e.g., Zubieta et al. 2003) and (sexual) pleasure (see Pfaus 2025), their respective role in SM‐related pleasurable experiences appears plausible. However, it has recently been found that changes in levels of endocannabinoids and cortisol following an SM‐interaction differ depending on the respective SM role (Wuyts et al. 2020). While submissive practitioners demonstrated increased levels of cortisol and endocannabinoids, dominant practitioners showed increased levels of endocannabinoids only when they engaged in power (Wuyts et al. 2020). This might suggest that for submissives, acute pain in the context of SM is both, stressful/thrilling (cortisol) and pleasurable (endocannabinoids), and that for dominants the interaction is only pleasurable when it is related to power dynamics (Wuyts et al. 2020).

In the literature, the distinction between the pleasantness of SM‐related and other types of pain in SM practitioners appears heterogenous: Defrin et al. (2015) found a negative correlation between pain in everyday life and masochistic behaviour, next to pain unpleasantness ratings that were comparable to the control group. However, ambiguously, practitioners still reported gaining significantly more pleasure from this type of pain than controls. A recent study by Forer and Westlake (2025) reported data on a positive relationship between SM and CP, suggesting short‐term pain relief, increased pain tolerance, a feeling of empowerment and other mental health benefits in SM practitioners with CP. Interestingly, they found a heightened CP prevalence of 41.9% in their sample, compared to around 30% reported in the general population (Steingrímsdóttir et al. 2017). The authors argue that more research is necessary to corroborate the observation as it might be accounted for by an unequal proportion of women in their sample, but did not test for sex effects. Importantly, they also did not include individuals without SM interest to control for biases that might have resulted in CP oversampling. To further investigate whether CP prevalence rates are increased in SM, we tested whether we could replicate the finding in a data set including individuals of both groups, originally collected to advance the validation of the *General Attitudes Towards Pain Inventory* (GATPI)—a psychometric instrument that we had previously developed and tested in SM and non‐SM (Vetterlein et al. 2022).

Accordingly, we further sought to replicate our earlier findings with respect to differences in general pain attitudes in individuals with and without SM interest (Vetterlein et al. 2022). In our previous study, participants with SM interest had been significantly more fascinated with pain, viewed pain more as a challenge, were more able to accept pain, and thought of pain less as a tragedy compared to participants without SM interest (Vetterlein et al. 2022).

Lastly, we were interested in identifying predictors of SM interest. Besides differences in pain attitudes, SM has priorly been associated with higher sensation seeking (Schuerwegen et al. 2021) and lower pain sensitivity (Defrin et al. 2015). More precisely, subjects engaging in masochistic behaviour have been found to show significantly higher pressure pain thresholds than controls (Defrin et al. 2015), although it should be noted that there is evidence that altered levels of pain sensitivity in masochists may depend on the respective context (Baudic et al. 2022; Kamping et al. 2016). We sought to analyse these variables, together with CP status, with respect to their predictive value, assuming that CP status, sensation seeking, pain fascination, viewing pain as a challenge and pain acceptance would be positive predictors and that pain sensitivity would be a negative predictor of SM.

## 2 Methods

The here‐reported study was in accordance with the Declaration of Helsinki. Participants completed an online survey presented via the online survey software *Unipark* (Tivian XI GmbH, Cologne, Germany). All measures were presented in German.

### 2.1 Participant Recruitment

Participants were primarily recruited in the university context via word‐of‐mouth advertisement, the student body's website, social media and flyers. To ensure a sufficiently large subsample of individuals with SM interest, we further contacted a German online dating platform with a large BDSM (bondage/discipline, dominance/submission and sadism/masochism) community. As incentive, participants could receive feedback on their subjective pain sensitivity. Psychology students could also receive course credit for participation. Before taking part in the study, participants gave informed consent.

### 2.2 Psychometric Instruments

To collect information on SM interest, all participants were asked whether they had a SM preference in an erotic sense (*yes/no*). If they agreed, they were further asked to indicate which of the following options they preferred: *active* (*dominant and/or sadistic*), *passive* (*submissive and/or masochistic*), or *both roles* (*switching*).

Participants' general attitudes towards pain were investigated by means of the GATPI (Vetterlein et al. 2022) measuring 10 different ‘inner tendencies towards pain and coping with pain, expressed by evaluations that might be of either positive or negative valence and might concern cognitive as well as affective and behavioural levels’ (Vetterlein et al. 2022, 3). Positive attitudes towards pain include being fascinated by pain and experiencing pain as pleasurable (*fascination‐pleasure*), accepting pain as a part of life (*acceptance‐stoicism*), perceiving pain as a challenge (*challenge*), as a valuable warning function (*warning*), or as something for which one can expect care and support by others (*secondary gain*). Negative attitudes towards pain subsume perceiving pain as something that no one should have to face (*tragedy*) or as a threat one should immediately tend to (*threat*), feeling helpless when facing pain (*helplessness*), experiencing pain as a punishment (*punishment*) or as an obstacle interfering with goal attainment (*obstacle*).

The GATPI is a 48‐item self‐report instrument with items such as ‘Pain has a certain appeal to me and somehow fascinates me’. (*fascination‐pleasure* subscale), ‘People who are in pain should avoid any exertion and let others do the work’. (*secondary gain* subscale), or ‘In my opinion, you do not have to endure pain—not even for a short time’. (*tragedy* subscale) to be answered on a five‐point Likert scale ranging from 1 (*strongly disagree*) to 5 (*strongly agree*). The inventory's psychometric properties have been extensively examined and its reliability and validity have been corroborated in a previous study (Vetterlein et al. 2022).

Next to pain attitudes, sensation seeking was measured by the *Sensation Seeking Scale Form V* (SSS‐V) (Beauducel et al. 2003; Zuckerman et al. 1964). The questionnaire consists of 40 items dichotomously measuring four aspects of sensation seeking: *thrill and adventure seeking* describes the desire for activities involving unusual sensations and risks (e.g., skydiving), *experience seeking* refers to new sensory or mental experiences through unconventional choices (e.g., travelling, drugs), *disinhibition* describes uncontrolled activities in social settings (e.g., partying, risky sexual behaviour) and *boredom susceptibility* is characterised by intolerance of repetition and boring social interactions, as well as a perceived restlessness in these situations. Adequate reliability and validity of the construct have been reported by multiple studies (Deditius‐Island and Caruso 2002; Zuckerman et al. 1964). For ethical reasons, item 22 (‘I would like to meet some persons who are homosexual (men or women)’ versus ‘I stay away from anyone I suspect of being “gay” *or* “*lesbian*.”’) was excluded in the present study, and item 29 (‘*I like to date members of the opposite sex who are physically exciting*’ versus ‘*I like to date members of the opposite sex who share my values*’) was generalised, both to avoid discrimination against people with non‐heteronormative sexual preferences.

Moreover, as a self‐report measure of pain sensitivity, the *Pain Sensitivity Questionnaire* (PSQ) (Ruscheweyh et al. 2009) was presented to the participants. The instrument consists of 14 items describing potentially painful everyday life situations to be rated on a numeric scale ranging from 0 (*not at all painful*) to 10 (*most severe pain imaginable*). Ruscheweyh and colleagues demonstrated adequate reliability of the PSQ and were able to validate the instrument by showing significant correlations between PSQ scores and experimental pain intensity ratings.

Lastly, participants were asked to provide information on their pain frequency (1 = *very rarely*; 2 = *multiple times per year*; 3 = *once a month*; 4 = *once a week*; 5 = *multiple times per week*; 6 = *daily*) and everyday pain‐related stress (1 = *none at all* to 7 = *extremely stressful*).

### 2.3 Statistical Analysis

*SPSS Statistics version 29* (IBM, Armonk, NY) was used for all statistical analyses if not indicated otherwise. The level of significance was set to *α* = 0.05. Mean scores were calculated for GATPI, SSS‐V and PSQ subscales. To control for sex and age difference between the non‐SM and SM sample, an age‐ and sex‐matched sample was created using the procedure established by Bacher (2002). All analyses were first conducted in the original, unmatched sample and then in the matched sample.

Chi‐squared tests were run to check for significant differences in CP prevalence between SM and non‐SM in the full sample and in female and male subsamples, as well as between SM roles. Exploratively, we tested for significant differences in pain frequency and everyday pain‐related stress between the non‐SM and SM sample by means of a Mann–Whitney *U* test and a *t*‐test for independent samples, respectively.

Beyond that, multiple ANOVAs were conducted to examine main and interaction effects of SM and CP on all GATPI subscales. Results of the Levene‐test were significant for the subscales *fascination‐pleasure, helplessness* and *punishment*; hence, we used the R package WRS2 (Mair and Wilcox 2020) to calculate robust ANOVAs for trimmed means (*γ* = 0.20).

Finally, we performed a hierarchical logistic regression (method: enter) with SM sexual preference (0 = *no*; 1 = *yes*) as criterion and the following blocks: *Pain status*, i.e., CP status (0 = *no*; 1 = *yes*) and PSQ total score (block 1), *personality*, that is, sensation seeking (block 2), and *pain attitudes*, that is, GATPI *challenge* and *acceptance & stoicism* (block 3). All variables were *z*‐standardised to allow for better comparison between the respective coefficients and odds ratios (OR). The blocks were added in ascending order, based on the assumed closeness of the constructs and the presumed variance explained by the predictors, to avoid early exclusion of relevant variables due to shared variance. We chose *challenge* and *acceptance & stoicism* as these are positive pain attitudes in which we had previously found higher scores in SM (Vetterlein et al. 2022). In a last step, we ran multiple logistic regressions entering all variables by themselves to examine the OR independent of the other variables. For sake of transparency, our initial regression analysis of the original sample included a block of demographic variables, which were not appropriate to include in the matched sample. Besides, we recognised that including the fascination‐pleasure subscale of the GATPI could be tautological and therefore excluded it from the analysis of the matched sample.

Exploratively, several post hoc moderation and mediation analyses (bootstrapping with 5000 samples; Davidson & MacKinnon heteroscedasticity consistent standard errors) were performed by means of the SPSS macro *PROCESS v4.2* (Hayes 2018), in order to explore reasons for these prevalence differences. In these models, we used CP as a predictor and SM sexual preference as a (dichotomous) criterion. We included pain‐related everyday stress in the model, once as a moderator and once as a mediator. Besides, we ran moderation analyses for the SSS‐V total score and the GATPI subscales *acceptance‐stoicism* and *helplessness*. Notably, all variables in the PROCESS models were *z*‐standardised to output standardised coefficients. We further ran a *t*‐test to test for significant differences in pain sensitivity (PSQ) between the non‐SM and SM sample.

Finally, we reran the logistic regression model, the mediation/moderation analyses, as well as the *t*‐test investigating differences in pain sensitivity again, thereby only including submissives and switchers in the SM‐subsample.

## 3 Results

### 3.1 Sample Characteristics

A total of *N* = 631 participants were originally recruited, 14 of which had to be excluded post hoc due to missing data (*N* = 2), being underaged (*N* = 5), incorrect answers provided in response to a check‐item (*N* = 6), and a repetitive response pattern (*N* = 1), resulting in final sample size of *N* = 617. The original sample's age ranged from 18 to 70 years with an average of *M* = 33.67 (SD = 13.47). 72.8% of the sample were female, 26.7% were male and 0.5% were intersex. Most participants had either completed a university degree (31.3%), a high school degree (43.1%), or vocational training (17.2%). 3.2% held a doctorate. 4.7% earned a middle school degree, one participant held an elementary school degree and two participants had not graduated from school. All participants indicated German to be their mother tongue. 94.5% were German citizens, 2.4% Austrian citizens, 3.1% indicated other citizenships. *N* = 242 participants (38.7%) reported to have an SM sexual preference, with 19.0% taking on an active/dominant role, 49.2% taking on a passive/submissive role, and 31.8% switching between both. The SM subsample was significantly (*t*_(615)_ = 9.57; *p* < 0.001; *d* = 0.79) older (*M* = 39.76; SD = 12.94) than the non‐SM subsample (*M* = 29.82; SD = 12.33). Besides, the subsamples significantly (*χ* ^2^_(1)_ = 16.96; *p* < 0.001; Cramér's *V* = 0.17) differed in sex distribution; however, both included more women (SM: 63.9%; non‐SM: 79.0%) than men (SM: 36.1% vs. non‐SM: 21.0%).

To be able to control for these differences, a matched sample was extracted which eventually consisted of *N* = 428 participants, 50% of which had an SM sexual preference, with 15% of these preferring an active/dominant role, 52.8% preferring a passive/submissive role, and 32.2% switching roles. Accordingly, the matched non‐SM and SM‐subsamples no longer significantly differed in age (non‐SM: *M* = 36.06; SD = 13.22; SM: *M* = 38.22; SD = 12.58; *t*_(426)_ = −1.73; *p* = 0.084; *d* = −0.17) or sex (non‐SM: 67.8% women and 32.2% men; SM: 69.2% women and 30.8% men; *χ* ^2^_(1)_ = 0.10; *p* = 0.755; Cramér's *V* = 0.15).

In the following, results will be presented for the matched sample. Results for the original sample can be found in the Supporting Information.

### 3.2 Chronic Pain in SM and Non‐SM

Table 1 offers cross tables and details with respect to the results of the Chi‐squared‐tests. A significantly higher proportion of participants with versus without SM sexual preference reported living with a CP condition. This was observed in the full sample as well as in female and male subsamples. There was no difference in the CP distribution between SM roles.

**TABLE 1:** Cross‐tables and results of the respective Chi‐squared‐tests examining chronic pain prevalence.

		CP		*χ* ^2^ (df)	*p*	Cramér's *V*
		No	Yes			
All (*N* = 428)				14.28 (1)	< 0.001	0.18
SM	No	151 (70.6)	63 (29.4)			
	Yes	113 (52.8)	101 (47.2)			
Women (*N* = 293)				4.85 (1)	0.028	0.13
SM	No	91 (62.8)	54 (37.2)			
	Yes	74 (50.0)	74 (50.0)			
Men (*N* = 135)				13.39 (1)	< 0.001	0.32
SM	No	60 (87.0)	9 (13.0)			
	Yes	39 (59.1)	27 (40.9)			
SM (*N* = 214)				1.09 (2)	0.579	0.07
Role	Dominant	16 (50.0)	16 (50.0)			
	Submissive	57 (50.4)	56 (49.6)			
	Switching	40 (58.0)	29 (42.0)			

*Note:* Frequencies are reported as cases (percentage).

Abbreviations: CP, chronic pain; SM, sadomasochistic sexual preference.

The distribution of pain frequencies differed significantly (*U* = 27,082.50; *Z* = 3.33; *p* < 0.001; *d* = 0.32) between the SM‐subsample (mdn = 3; IQR = 3) and in the non‐SM‐subsample (mdn = 3; IQR = 2). However, the groups did not differ in their pain‐related everyday stress burden (non‐SM: *M* = 2.60; SD = 1.95; SM: *M* = 2.92; SD = 1.92; *t*_(426)_ = −1.70; *p* = 0.090; *d* = −0.16).

### 3.3 Differences in General Attitudes Towards Pain in SM and CP

**FIGURE 1:**
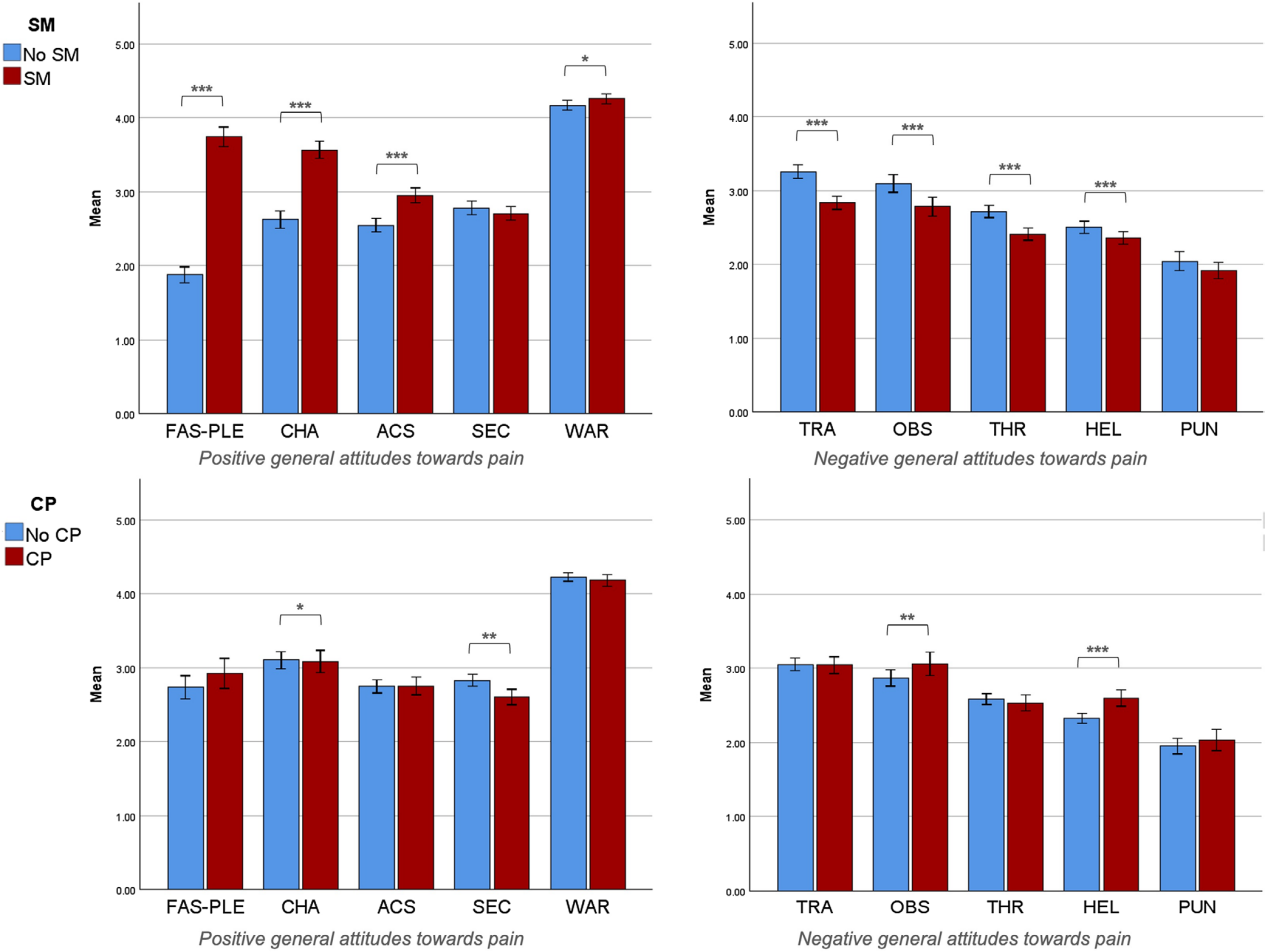
Main effects of sadomasochistic sexual preference (SM) and chronic pain (CP) status. *N* = 428. ACS, acceptance & stoicism; CHA, challenge; FAS‐PLE, fascination‐pleasure; HEL, helplessness; OBS, obstacle; PUN, punishment; SEC, secondary gain; THR, threat; TRA, tragedy; WAR, warning function. Subscales are sorted ascendingly by effect size for SM. ***p < 0.001; **p < 0.01; *p < 0.05.

While the ANOVAs revealed multiple main effects of both, SM and CP, on pain attitudes (see Table 2; Figure 1), there were no significant interaction effects of SM × CP. Participants with SM sexual preference generally demonstrated significantly stronger positive attitudes towards pain and significantly weaker negative pain attitudes than those without. An exception of this was seen with respect to the GATPI subscales *secondary gain* and *punishment*, where there were no significant differences. Effect sizes were largest for *fascination‐pleasure, challenge*, *acceptance & stoicism* and *tragedy*, explaining 53%, 24% and 8% of the variance, respectively. Differences in pain attitudes between SM roles can be found in the Supporting Information.

**TABLE 2:** Main and interaction effects of SM and CP on GATPI subscales.

Subscale		*M* (SD)_yes_	*M* (SD)_no_	*F*_(1,424)_	*p*	*η* ^2^
FAS‐PLE	SM	3.74 (0.98)	1.88 (0.78)	535.06	**0.001**	0.53
	CP	2.92 (1.30)	2.74 (1.28)	3.14	0.078	0.01
	SM × CP			0.35	0.555	0.00
CHA	SM	3.57 (0.84)	2.63 (0.85)	135.43	**< 0.001**	0.24
	CP	3.09 (0.97)	3.10 (0.96)	5.79	**0.017**	0.01
	SM × CP			0.36	0.547	0.00
WAR	SM	4.26 (0.49)	4.17 (0.52)	4.26	**0.040**	0.01
	CP	4.18 (0.53)	4.23 (0.49)	1.77	0.183	0.00
	SM × CP			0.22	0.636	0.00
ACS	SM	2.95 (0.73)	2.55 (0.70)	37.53	**< 0.001**	0.08
	CP	2.76 (0.77)	2.75 (0.73)	1.23	0.269	0.00
	SM × CP			2.65	0.104	0.01
SEC	SM	2.71 (0.68)	2.78 (0.69)	0.05	0.827	0.00
	CP	2.60 (0.68)	2.83 (0.68)	10.90	**0.001**	0.03
	SM × CP			1.17	0.280	0.00
TRA	SM	2.84 (0.67)	3.26 (0.68)	36.84	**< 0.001**	0.08
	CP	3.04 (0.73)	3.05 (0.70)	0.89	0.347	0.00
	SM × CP			1.17	0.280	0.00
THR	SM	2.41 (0.62)	2.72 (0.62)	22.04	**< 0.001**	0.05
	CP	2.53 (0.68)	2.59 (0.61)	0.00	0.955	0.00
	SM × CP			0.31	0.579	0.00
HEL	SM	2.36 (0.64)	2.50 (0.62)	9.89	**0.003**	0.03
	CP	2.60 (0.70)	2.33 (0.56)	19.57	**0.001**	0.06
	SM × CP			0.67	0.415	0.00
PUN	SM	1.92 (0.80)	2.05 (0.97)	1.12	0.294	0.01
	CP	2.04 (0.94)	1.95 (0.86)	1.01	0.218	0.01
	SM × CP			1.46	0.231	0.00
OBS	SM	2.79 (0.96)	3.10 (0.90)	16.53	**< 0.001**	0.04
	CP	3.06 (1.01)	2.87 (0.89)	7.96	**0.005**	0.02
	SM × CP			0.77	0.381	0.00

*Note:* *N* = 428. Effect sizes for FAS‐PLE, HEL and PUN were estimated without trimmed means to reflect the full sample. Significant *p*‐values are printed in bold.

Abbreviations: ACS, acceptance & stoicism; CHA, challenge; CP, chronic pain; FAS‐PLE, fascination‐pleasure; HEL, helplessness; OBS, obstacle; PUN, punishment; SEC, secondary gain; SM, sadomasochistic sexual preference; THR, threat; TRA, tragedy; WAR, warning function.

The effect of CP on pain attitudes was rather small (≤ 5%). Participants with CP showed significantly lower *challenge* and *secondary gain* scores but scored significantly higher on *helplessness* and *obstacle* than participants without CP.

### 3.4 Predictors of SM Sexual Preference

Entering CP status and pain sensitivity (model 1) into a hierarchical logistic regression model predicting SM sexual preference explained around 5% of the variance, although only CP status yielded significance. The addition of sensation seeking (model 2) led to a 19% increase in the variance explained, that is, 24% in total. In a last step, we added the GATPI subscales *challenge*, and *acceptance & stoicism* (model 3). Of the two pain attitudes, *challenge* was the only significant predictor. The final model was able to explain 42% of the observed variance and was able to correctly classify 75.2% of the participants with respect to SM; suggesting that CP, higher sensation seeking and viewing pain as a challenge jointly contribute to SM sexual preference (see Table 3 for full details on all models).

**TABLE 3:** Hierarchical logistic regression predicting SM sexual preference.

Model	Predictor	% correctly classified	Nagelkerke *R* ^2^	*β*	SE	OR	Wald	*p*
0	Constant	**50.0**	—					
				< 0.01	0.10	1.00	< 0.01	> 0.99
1		**58.9**	**0.05**					
	Constant			< 0.01	0.09	1.00	< 0.01	0.983
	CP status			0.38	0.10	1.46	14.45	**< 0.001**
	PSQ total			−0.10	0.10	0.90	1.06	0.304
2		**67.1**	**0.24**					
	Constant			< 0.01	0.11	1.00	< 0.01	0.993
	CP status			0.53	0.11	1.69	22.43	**< 0.001**
	PSQ total			−0.03	0.11	0.97	0.06	0.809
	SSS‐V total			0.93	0.13	2.53	54.98	**< 0.001**
3		**75.2**	**0.42**					
	Constant			−0.01	0.12	0.99	0.01	0.911
	CP status			0.58	0.12	1.79	22.40	**< 0.001**
	PSQ total			0.05	0.12	1.05	0.15	0.696
	SSS‐V total			0.14	0.14	1.93	23.45	**< 0.001**
	GATPI CHA			1.07	0.15	2.91	49.82	**< 0.001**
	GATPI ACS			0.11	0.13	1.12	0.72	0.397

*Note:* *N* = 428. SM sexual preference: 0 = no, 1 = yes. CP status: 0 = no, 1 = yes. Significant *p*‐values are printed in bold.

Abbreviations: ACS, acceptance & stoicism; CHA, challenge; CP, chronic pain; GATPI, General Attitudes Towards Pain Inventory; OR, odds ratio; PSQ, Pain Sensitivity Questionnaire; SSS‐V, Sensation Seeking Scale Form V.

**FIGURE 2:**
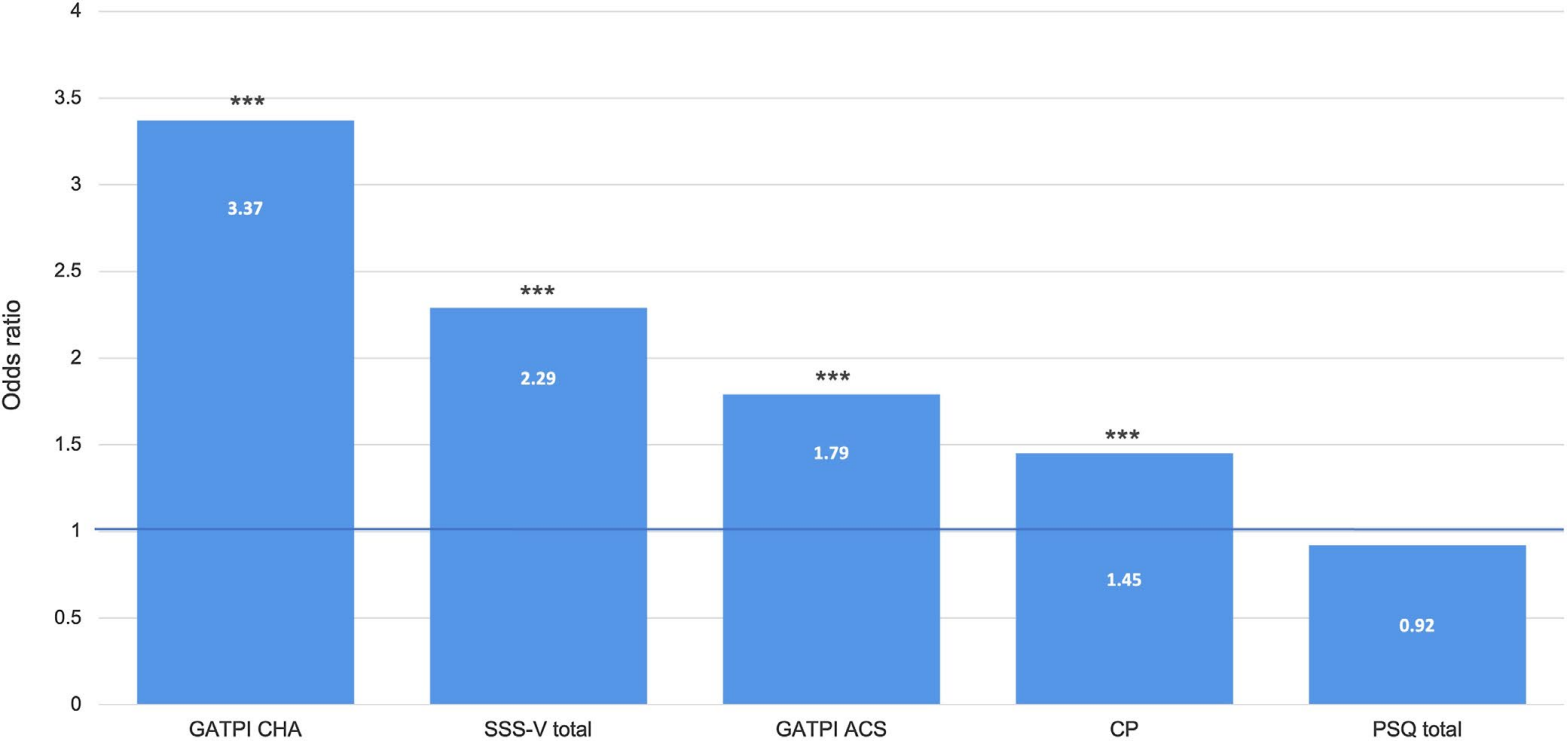
Odds ratios of variables predicting sadomasochistic sexual preference (in descending order). ACS, acceptance & stoicism; CHA, fascination‐pleasure; CP, chronic pain; GATPI, General Attitudes Towards Pain Inventory; PSQ, Pain Sensitivity Questionnaire; SSS‐V, Sensation Seeking Scale Form V. Significance of predictors: ***p < 0.001.

To be able to look at the respective OR independent of shared variance, we re‐entered each variable into the model separately (see Figure 2). *Challenge* presented the highest OR, with an around three‐fold increase in probability. This was followed by sensation seeking, where the probability was more than doubled, and *acceptance‐stoicism* with an OR of 1.79. CP patients were around 1.5 times as likely to show an SM sexual preference. Pain sensitivity was not a significant predictor.

### 3.5 Explorative Analyses

Pain‐related everyday stress was neither a significant mediator (ab: −0.02; 95%‐CI [−0.14, 0.10]) nor a moderator (*β*_Int_ = −0.02; *p* = 0.861) in the relationship between CP and SM. Likewise, neither sensation seeking (*β*_Int_ = 0.16; *p* = 0.223) nor pain acceptance (*β*_Int_ = 0.17; *p* = 0.137) nor pain helplessness (*β*_Int_ = −0.03; *p* = 0.778) was found to be moderators.

There was no significant difference (*t*_(426)_ = 0.81; *p* = 0.419; *d* = 0.08) in pain sensitivity between the non‐SM (*M* = 3.96; SD = 1.48) and SM (*M* = 3.84; SD = 1.42) subsamples.

## 4 Discussion

### 4.1 Is CP Prevalence Higher in SM?

We were able to replicate the finding by Forer and Westlake (2025), which had reported a CP prevalence of 41.9% in individuals with SM sexual preference. Similar to the authors, we found a substantially increased prevalence of 47.2%, compared to the numbers usually reported of around 30% (Steingrímsdóttir et al. 2017). In contrast to Forer and Westlake (2025), our sample included a control group, demonstrating an expectable CP prevalence of 29.4%. This rules out the idea brought forward by Forer and Westlake (2025) that self‐identification as CP patient could explain the heightened prevalence. Since Forer and Westlake (2025) further argued that it might have been a result of oversampling women, we used an age‐ and sex‐matched sample and analysed both sexes separately. Although—in line with the literature (Steingrímsdóttir et al. 2017)—we did find higher CP prevalences in women (SM: 50.0%; non‐SM: 37.2%) than in men (SM: 40.9%; non‐SM: 13.0%), we found the increase in the SM sample irrespective of sex. Moreover, with respect to the matched sample, the effect does not seem to be attributed to higher age in both CP and SM. It should be noted, however, that controlling for sex and age clearly does not render our sample representative, and large epidemiological investigations should still be conducted to corroborate the result's validity, for example, in subgroups with lower education.

Of note, the effect does not seem to depend on SM‐roles either, seeing that we did not find any significant differences in CP prevalence rates between dominants, submissives and switchers.

### 4.2 Why Would CP Prevalence Be Increased?

The reasons underlying higher CP prevalence rates in SM remain elusive. In our data, pain‐related everyday stress neither seemed to mediate nor to moderate the relationship. Similarly, sensation seeking, pain acceptance and pain helplessness were not found to be significant moderators. These results held when we reran the analyses only including submissives and switchers, which would actually receive painful stimulation (see Supplementary Analyses).

As there were no significant differences in pain sensitivity between the non‐SM and SM subsamples, it further seems unlikely that the heightened CP prevalences are related to this. Since masochists, specifically, had previously been found to show significantly lower pain thresholds (Defrin et al. 2015), we reran the analysis again, only including submissives and switchers in the SM subsample (see Supporting Information). Still, no significant differences in pain sensitivity were found. Potentially, the effect is only seen in objective pain sensitivity markers as recorded in quantitative sensory testing in the laboratory.

Based on our data, we cannot specify the directionality of the SM‐CP relationship. We cannot rule out the fact that SM interest could precede CP; however, lower pain thresholds in masochists would rather speak to the inverse directionality, seeing that pain sensitivity is thought to be a risk factor for CP (Nielsen et al. 2009). On the other hand, a common personality in SM and CP could possibly mediate the relationship, as in both, SM and CP, there is preliminary evidence for increased prevalences in personality disorders (Conrad et al. 2007; De Neef et al. 2019). Yet, SM has been associated with lower neuroticism and more secure attachment styles (Wismeijer and van Assen 2013), while CP has been related to higher neuroticism and insecure attachment (Naylor et al. 2017). Hence, it could be a fruitful approach to ask the other way around: Why could CP lead to SM interest?

Based on our data we cannot explain which CP patients are more likely to engage in SM than others. Although we could replicate and extend previously found main effects of SM on pain attitudes (Vetterlein et al. 2022) and could present first evidence of the GATPI's validity in CP, we did not find significant interaction effects between CP and SM that could have helped gain a deeper understanding of the subgroup with CP *and* SM sexual preference. Previous literature, however, points to the use of SM practise as a coping strategy for CP: The experience of acute pain in the context of SM might override CP (i.e., fighting pain with pain) and serve as a short‐term pain relief, likely mediated through the release of endogenous opioids in response to acute noxious stimulation (Brown, Iverson, and Cari 2020; Dunkley et al. 2020; Forer and Westlake 2025). SM has further been found to relief emotional distress (Duarte Silva 2015; Schuerwegen et al. 2021) and to induce flow‐like states of altered consciousness, increased bodily awareness, and mindfulness (Ambler et al. 2017; Bastian, Jetten, Hornsey, and Leknes 2014; Carlström 2021; Dunkley et al. 2020). SM practitioners with CP further report self‐empowerment (Forer and Westlake 2025), regaining some form of control over pain (Jobson 2020), strengthening their pain tolerance (Forer and Westlake 2025) and feeling less disabled (Sheppard 2018), suggesting an improvement in pain‐related self‐efficacy through SM practise. In this sense, SM practise could be a means to reframing pain. Socially, CP patients who practise SM share pain with others as part of an interdependent relationship, instead of relying on others for help (Jobson 2020). Seeing that we did not find significant differences between SM roles, it appears plausible to assume that several coping mechanisms are involved, as not all of them apply to each role.

Notably, many of the ideas presented here come from qualitative interviews and case studies. Going forward, quantitative studies in larger samples are desirable, particularly prospective studies shedding more light on the directionality of the relationship. As for now, the idea of SM as a coping strategy appears convincing and is supported by previous evidence demonstrating a generally higher use of active coping strategies in SM practitioners and 40% reporting using SM for coping (Schuerwegen et al. 2021). Besides, increased rates of non‐suicidal self‐injury (where painful stimulation is used to provide stress relief; Franklin et al. 2010) have also been reported in CP and have been interpreted as a means for self‐regulation (Johnson et al. 2022). In a similar vein, there is preliminary evidence for higher prevalence rates of borderline personality disorder in individuals with SM interest (Frías et al. 2017), as well. Seemingly, acute physical pain can provide short‐term relief from both emotional and chronic physical pain.

### 4.3 Who Is More Likely to Show SM Sexual Preference in General?

We have identified pain‐related and psychological factors predicting SM sexual preference, together explaining around 42% of the variance and accurately classifying 75.2% of the participants. According to these results, individuals with CP and higher sensation seeking who view pain as a challenge are most likely to engage in SM. Of note, the results held when we reran the regression analyses and only included submissives and switchers in the SM subsample (see Supporting Information), suggesting that these findings are not dependent on SM role.

Sensation seeking as a predictor of SM is supported by prior literature (Schuerwegen et al. 2021; Vetterlein et al. 2022). It is thought that the desire for intense stimulation and openness for new and exciting experiences may be what leads to an increased interest in SM (De Neef et al. 2019; Schuerwegen et al. 2021), which is further underlined by evidence of higher openness in individuals with SM interest (Paarnio et al. 2023).

Notably, viewing pain as a challenge added 18% of explained variance to our model. This is in line with previous literature (Vetterlein et al. 2022). High scores can also be found in individuals who practise high‐risk sports (Vetterlein et al. unpublished data) and would be expected to be found in individuals with a preference for pungent food (see Defrin et al. 2021). Hence, SM sexual preference could reflect a tendency to test one's own boundaries.

Interestingly, when conducting a similar regression analysis in the non‐matched original sample, including demographic variables, a positive relationship between age and SM was found (see Supporting Information). This goes against previous literature (Brown, Barker, and Rahman 2020; Paarnio et al. 2023). As the majority of our SM subsample had been recruited via an online dating platform operating since 2005, the community addressed there can be assumed to have been somewhat older than the student population from which the majority of the non‐SM sample presumably had been recruited, potentially serving as an explanation. However, it could also be argued that the longer an individual has lived, the greater the likelihood of their exposure to SM.

Moreover, in the analysis of the original, unmatched sample, sex almost reached significance in the final model (*p* < 0.052; see Supporting Information), with men showing a slightly higher likelihood. Thus, a closer investigation of sex differences appears intriguing. Previous literature did not find such disparities, yet a significant difference in roles, with women being more likely to take on submissive roles (Brown, Barker, and Rahman 2020). Post hoc analyses of our data replicated this (see Supporting Information), potentially mirroring internalised patriarchal structures (Brown, Barker, and Rahman 2020).

### 4.4 General Strengths and Limitations

To our best knowledge, this is the first study to thoroughly investigate SM sexual preference together with CP status, pain sensitivity, sensation seeking and pain attitudes, thereby contributing to the growing body of quantitative psychological research on SM. The sample was quite large, especially with respect to the SM subsample, and served to replicate and extend the findings by Forer and Westlake (2025). As per usual in psychological research, less than a third of the original sample was male, resulting in a disproportionate sex distribution. However, using an age‐ and sex‐matched sample we could control for potential sex effects, which Forer and Westlake (2025) had not. Moreover, the inclusion of a non‐SM subsample allowed for a comparison, thereby controlling for biases resulting in an oversampling of CP, as discussed above. In a similar vein, next to the recruitment within said online dating platform, the most prominent underlying population from which participants were recruited was students who took part for course credit, potentially leading to a younger age in the non‐SM subgroup in the original sample. This could present an issue when regarding CP prevalence in SM, in light of the known positive relationship between age and CP (Tinnirello et al. 2021). Yet, again, by using a matched sample, we were able to control for potential influences.

The arguably most important limitation of our study is its cross‐sectional approach, not allowing for causal inference and, hence, leaving the open question as to why CP prevalence rates might be elevated in SM: Does SM increase the risk of developing CP or does SM serve as a coping strategy in CP?

### 4.5 Further Implications and Future Directions

Our study's findings add to the understanding of SM interest as well as its correlates and may inform future research in the field. Most importantly, longitudinal studies are needed to shed light on the directionality of the SM‐CP relationship.

If SM is demonstrated to precede CP, it would be fruitful to further assess possible psychological and biological mediators. Previous literature suggests a dysregulation in the brain's reward system mediated by altered levels of dopamine and opioids in both CP and non‐suicidal self‐harm (Johnson et al. 2022; Massaly et al. 2016). These neurotransmitters have also been previously discussed to play a role in SM sexual preference (Wuyts and Morrens 2022). Moreover, sensation seeking, which we found to predict SM interest, has previously been associated with a hyper‐reactive dopamine system (Norbury and Husain 2015), leading to the question if differences in tonic levels of dopamine (and opioids?) could be a common denominator in the relationship between CP and SM.

If future studies confirmed the idea that SM can serve as a coping strategy, it would be interesting to see whether it is even adaptive for those patients practising it, and whether it is more adaptive for certain CP conditions than for others. When transferring these ideas to general CP treatment, this might not exactly mean suggesting SM in pain therapy. There could, however, be lessons to learn in terms of its beneficial effects. These could relate to improving pain‐related self‐efficacy, reframing the meaning of pain, differentiating acute and chronic pain, and developing more mutual social relationships.

### 4.6 Conclusion

We reported further evidence on pain as a pleasurable experience and presented data on the unexpected relationship between SM and CP. We demonstrated elevated CP prevalence in SM and found a predictive model of SM including CP, sensation seeking and viewing pain as a challenge, explaining around 42% of the variance. Future studies are necessary to corroborate the idea of SM as a coping strategy potentially used by some CP patients to gain the upper hand.

## Author Contributions

All authors contributed to the conception of the study, discussed the results and their interpretations, revised and approved the article. A.V., S.K. and M.M. performed the statistical analyses. A.V. and S.K. drafted the article and designed the figures.

## Funding

The authors have nothing to report.

## Conflicts of Interest

The authors declare no conflicts of interest.

## Supporting information


**Data S1:** ejp70230‐sup‐0001‐Supinfo01.docx.

## Data Availability

The data that support the findings of this study are available from the corresponding author, A.V., upon reasonable request.
