# Supplementary material for: Gaining the Upper Hand? Further Evidence of Pain as a Pleasurable Experience and the Unexpected Relationship Between Sadomasochistic Sexual Preference and Chronic Pain
Source: Eur J Pain. 2026 Feb 7;30(2):e70230. doi: 10.1002/ejp.70230 (PMC12882782; doi:10.1002/ejp.70230)
Supplement: Supplementary file 1 — Data S1: ejp70230‐sup‐0001‐Supinfo01.docx. [file EJP-30-0-s001.docx]

**Supplementary Materials (Vetterlein et al.)**

**A. Results (original, non-matched sample)**

**Table A**. Cross-tables and results of the respective Chi^2^-tests examining chronic pain prevalence.

|  |  | CP | | |  |  |  |
| --- | --- | --- | --- | --- | --- | --- | --- |
|  |  | *No* | | *Yes* | χ*^2^ (df)* | *p* | *Cramér's V* |
| **All (*N* = 617)** | | |  |  | **24.43 (1)** | **< .001** | **.20** |
| SM | *No* | | 280 (74.1) | 98 (25.9) |  |  |  |
|  | *Yes* | | 131 (54.8) | 108 (45.2) |  |  |  |
| **Women (*N* = 449)** | | |  |  | **20.48 (1)** | **< .001** | **.21** |
| SM | *No* | | 211 (71.0) | 86 (29.0) |  |  |  |
|  | *Yes* | | 75 (49.3) | 77 (50.7) |  |  |  |
| **Men (*N* = 165)** | | |  |  | **12.06 (1)** | **< .001** | **.27** |
| SM | *No* | | 69 (87.3) | 10 (12.7) |  |  |  |
|  | *Yes* | | 55 (64.0) | 31 (36.0) |  |  |  |
| **SM (*N* = 242)** | | |  |  | **0.95 (1)** | **.621** | **.06** |
| Role | *Dominant* | | 27 (61.4) | 17 (38.6) |  |  |  |
|  | *Submissive* | | 63 (52.9) | 56 (47.1) |  |  |  |
|  | *Switching* | | 41 (53.9) | 35 (46.1) |  |  |  |

*Note.* SM = Sadomasochistic sexual preference. CP = Chronic pain. Frequencies are reported as cases (percentage).

In line with the results of the Chi^2^-tests, there was a small significant positive point biserial correlation between SM sexual preference and CP status (*r_pb_* = .20; *p* < .001), which held when age was controlled for (*r_pb_* = .12; *p* = .002). Pain-related everyday stress was neither a significant mediator (ab: -.03; 95%-CI [-.13, .07]) nor moderator (β_Int_ = -.09; *p* = .374) in the relationship between CP and SM. Likewise, neither sensation seeking (β_Int_ = .01; *p* = .900), nor pain acceptance (β_Int_ = .12; *p* = .210), nor pain helplessness (β_Int_ = .02; *p* = .829) were found to be moderators. There was no significant difference (*t*_(615)_ = 1.04; *p* = .300; *d* = 0.09) in pain sensitivity between the non-SM (*M* = 3.97; *SD* = 1.44) and SM (*M* = 3.85; *SD* = 1.40) subsamples.

**Table B**. Main and interaction effects of SM and CP on GATPI subscales.

| Subscale |  | *M (SD)_yes_* | *M (SD)_no_* | *F _(1, 613)_* | *p* | η² |
| --- | --- | --- | --- | --- | --- | --- |
| FAS-PLE | SM | 3.71 (1.02) | 1.95 (0.79) | 533.77 | **< .001** | .47 |
|  | CP | 2.79 (1.27) | 2.56 (1.21) | 2.58 | .076 | .01 |
|  | SM x CP |  |  | 0.00 | .930 | .00 |
| CHA | SM | 3.56 (0.85) | 2.72 (0.82) | 142.06 | **< .001** | .19 |
|  | CP | 3.06 (0.93) | 3.04 (0.92) | 4.54 | **.034** | .01 |
|  | SM x CP |  |  | 0.03 | .869 | .00 |
| WAR | SM | 4.26 (0.48) | 4.16 (0.52) | 7.26 | .**007** | .01 |
|  | CP | 4.17 (0.53) | 4.21 (0.50) | 2.31 | .129 | .00 |
|  | SM x CP |  |  | 0.31 | .576 | .00 |
| ACS | SM | 2.96 (0.72) | 2.56 (0.67) | 53.72 | **< .001** | .08 |
|  | CP | 2.72 (0.76) | 2.71 (0.70) | 1.28 | .259 | .00 |
|  | SM x CP |  |  | 2.61 | .107 | .00 |
| SEC | SM | 2.71 (0.67) | 2.92 (0.68) | 6.66 | **.010** | .01 |
|  | CP | 2.67 (0.72) | 2.92 (0.65) | 13.01 | **< .001** | .02 |
|  | SM x CP |  |  | 1.53 | .217 | .00 |
| TRA | SM | 2.84 (0.67) | 3.29 (0.67) | 54.89 | **< .001** | .08 |
|  | CP | 3.09 (0.75) | 3.13 (0.68) | 1.35 | .246 | .00 |
|  | SM x CP |  |  | 2.30 | .130 | .00 |
| THR | SM | 2.43 (0.64) | 2.74 (0.67) | 26.26 | **< .001** | .04 |
|  | CP | 2.58 (0.71) | 2.64 (0.64) | 0.02 | .884 | .00 |
|  | SM x CP |  |  | 0.52 | .470 | .00 |
| HEL | SM | 2.35 (0.63) | 2.53 (0.65) | 20.44 | **< .001** | .03 |
|  | CP | 2.63 (0.73) | 2.37 (0.59) | 29.82 | **< .001** | .05 |
|  | SM x CP |  |  | 0.61 | .435 | .00 |
| PUN | SM | 1.93 (0.81) | 2.06 (0.91) | 4.96 | **.026** | .01 |
|  | CP | 2.07 (0.92) | 1.98 (0.85) | 2.18 | .141 | .00 |
|  | SM x CP |  |  | 0.39 | .532 | .00 |
| OBS | SM | 2.75 (0.94) | 3.14 (0.87) | 34.88 | **< .001** | .05 |
|  | CP | 3.13 (0.98) | 2.92 (0.87) | 15.49 | **< .001** | .03 |
|  | SM x CP |  |  | 0.35 | .555 | .00 |

*Note.* *N* = 617. SM = Sadomasochistic sexual preference. CP = Chronic pain. FAS-PLE = Fascination-Pleasure. CHA = Challenge. WAR = Warning Function. ACS = Acceptance & Stoicism. SEC = Secondary Gain. TRA = Tragedy. THR = Threat. HEL = Helplessness. PUN = Punishment. OBS = Obstacle. Effect sizes for FAS-PLE and SEC were estimated without trimmed means to reflect the full sample. Significant *p*-values are printed in bold.

**Table C**. Hierarchical logistic regression predicting SM sexual preference.

| Model | Predictor | % correctly classified | Nagelkerke  R^2^ | | β | *SE* | *OR* | *Wald* | *p* |
| --- | --- | --- | --- | --- | --- | --- | --- | --- | --- |
| **0** | Constant | **61.2** | - | |  |  |  |  |  |
|  |  |  |  | | -0.46 | 0.08 | 0.63 | 30.48 | < .001 |
| **1** |  | **67.6** | **.18** | |  |  |  |  |  |
|  | Constant |  |  | | -0.50 | 0.09 | 0.61 | 43.07 | < .001 |
|  | Sex |  |  | | 0.19 | 0.09 | 2.13 | 4.33 | **.038** |
|  | Age |  |  | | 0.76 | 0.10 | 0.93 | 59.33 | **< .001** |
|  | Education |  |  | | -0.08 | 0.10 | 0.93 | 0.69 | .408 |
| **2** |  | **68.2** | **.20** | |  |  |  |  |  |
|  | Constant |  |  | | -0.51 | 0.09 | 0.60 | 26.49 | < .001 |
|  | Sex |  |  | | 0.24 | 0.09 | 1.27 | 6.47 | **.011** |
|  | Age |  |  | | 0.69 | 0.10 | 1.99 | 44.87 | **< .001** |
|  | Education |  |  | | -0.06 | 0.10 | 0.94 | 0.43 | .514 |
|  | CP status |  |  | | 0.31 | 0.09 | 1.38 | 12.04 | **< .001** |
|  | PSQ total |  |  | | -0.15 | 0.09 | 0.87 | 2.52 | .113 |
| **3** |  | **72.6** | **.33** | |  |  |  |  |  |
|  | Constant |  |  | | -0.59 | .10 | 0.56 | 63.33 | < .001 |
|  | Sex |  |  | | 0.08 | .11 | 1.08 | 0.55 | .458 |
|  | Age |  |  | | 0.79 | .10 | 2.21 | 52.50 | **< .001** |
|  | Education |  |  | | < 0.01 | .10 | 1.00 | < 0.01 | .974 |
|  | CP status |  |  | | 0.38 | .10 | 1.46 | 14.76 | **< .001** |
|  | PSQ total |  |  | | -0.09 | .11 | 0.91 | 0.88 | .348 |
|  | SSS-V total |  | |  | 0.83 | .10 | 2.29 | 58.22 | **< .001** |
| **4** |  | **87.3** | **.69** | |  |  |  |  |  |
|  | Constant |  |  | | -0.83 | 0.14 | 0.44 | 65.67 | < .001 |
|  | Sex |  |  | | 0.27 | 0.14 | 1.31 | 3.76 | .052 |
|  | Age |  |  | | 0.80 | 0.14 | 2.23 | 31.14 | **< .001** |
|  | Education |  |  | | 0.16 | 0.14 | 1.17 | 1.28 | .258 |
|  | CP status |  |  | | 0.50 | 0.14 | 1.64 | 13.08 | **< .001** |
|  | PSQ total |  |  | | -0.10 | 0.13 | 0.90 | 0.58 | .448 |
|  | SSS-V |  |  | | 0.32 | 0.15 | 1.37 | 4.70 | **.030** |
|  | GATPI FAS-PLE |  |  | | 2.30 | 0.23 | 9.98 | 102.83 | **< .001** |
|  | GATPI CHA |  |  | | -0.18 | 0.20 | 0.83 | 0.87 | .352 |
|  | GATPI ACS |  |  | | 0.10 | 0.16 | 1.01 | 0.01 | .945 |

*Note.* *N* = 614. *df* = 1. OR = Odds ratio. CP = Chronic pain. PSQ = Pain Sensitivity Questionnaire. SSS-V = Sensation Seeking Scale Form V. GATPI = General Attitudes Towards Pain Inventory. FAS-PLE = Fascination-Pleasure. CHA = Challenge. ACS = Acceptance & Stoicism. Sex: 0 = female, 1 = male. CP status: 0 = no, 1 = yes. Significant *p*-values are printed in bold.


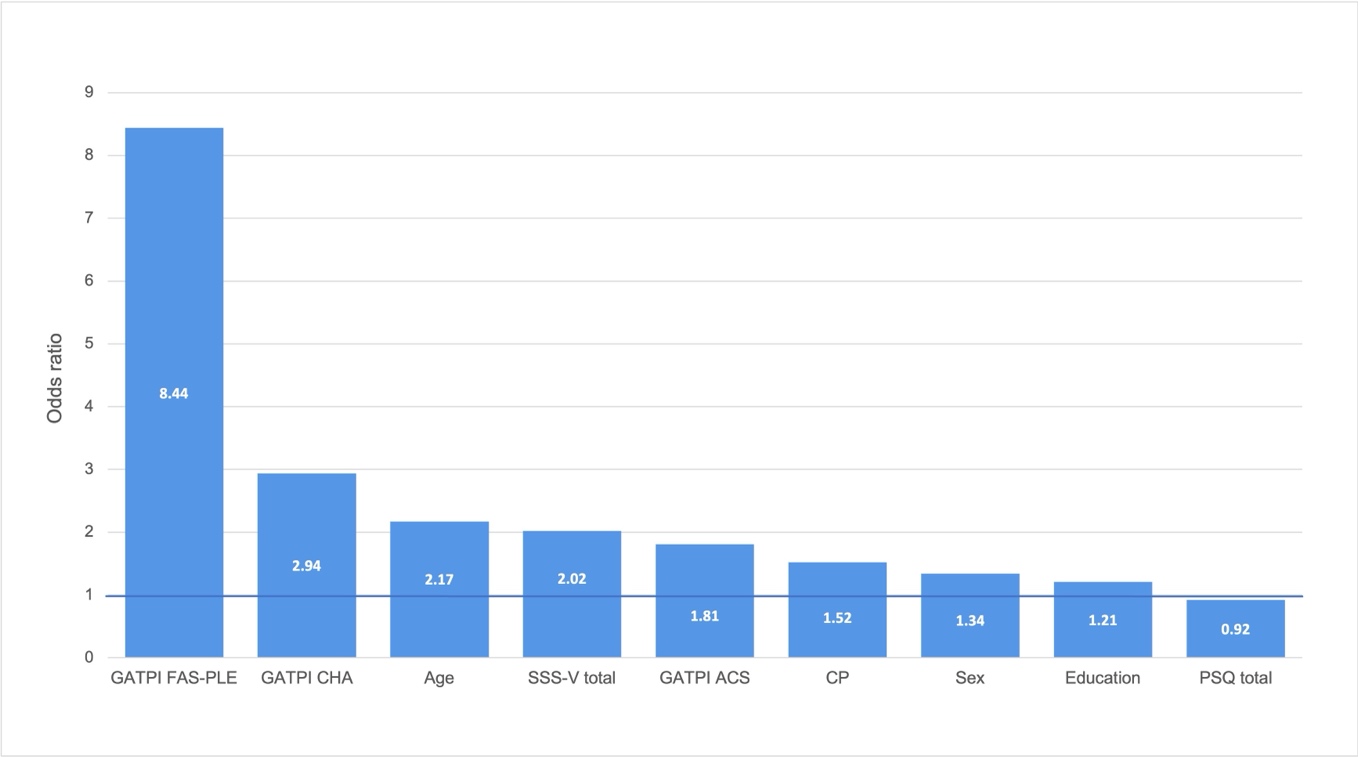


**Figure A**. Odds ratios of variables predicting sadomasochistic sexual preference (in descending order). GATPI = General Attitudes Towards Pain Inventory. FAS-PLE = Fascination-Pleasure. CHA = Challenge. ACS = Acceptance & Stoicism. CP = Chronic pain. SSS-V = Sensation Seeking Scale Form V. PSQ = Pain Sensitivity Questionnaire. Significance of predictors: *** *p* < .001; * *p* < .05.

**Table D**. Sex differences in SM roles.

|  |  | Sex | |  |  |  |
| --- | --- | --- | --- | --- | --- | --- |
|  |  | *Female* | *Male* | χ*^2^ (df)* | *p* | *Cramér‘s V* |
| **SM (*N* = 242)** | |  |  | **61.03 (2)** | **< .001** | **.51** |
| Role | *Dominant* | 8 (5.3) | 36 (41.9) |  |  |  |
|  | *Submissive* | 99 (65.1) | 19 (22.1) |  |  |  |
|  | *Switching* | 45 (29.6) | 31 (36.0) |  |  |  |

*Note*. SM = Sadomasochistic sexual preference. Frequencies are reported as cases (percentage).

**B. Explorative analyses (matched sample)**

When rerunning the analyses with only submissives and switchers included in the SM subsample, pain-related everyday stress was neither a significant mediator (ab: -.04; 95%-CI [-.16, .09]) nor a moderator (β_Int_ = < -.01; *p* = .991) in the relationship between CP and SM. Likewise, neither sensation seeking (β_Int_ = .15; *p* = .287), nor pain acceptance (β_Int_ = .18; *p* = .143), nor pain helplessness (β_Int_ = -.06; *p* = .606) were found to be moderators. There was no significant difference (*t*_(394)_ = 0.87; *p* = .383; *d* = 0.09) in pain sensitivity between the non-SM (*M* = 3.96; *SD* = 1.48) and SM (*M* = 3.83; *SD* = 1.40) subsamples.

**Table E**. Mean differences (ANOVA) between the three SM role preferences in GATPI subscales.

| Subscale | Role | *M* | *SD* | *F* | *p* | η² |  | Subscale | Role | *M* | *SD* | *F* | *p* | η² |
| --- | --- | --- | --- | --- | --- | --- | --- | --- | --- | --- | --- | --- | --- | --- |
| **FAS-PLE** |  |  |  | 37.13 | **<.001** | .26 |  | **TRA** |  |  |  | 0.98 | .379 | .01 |
|  | Dominant ^a^ | 2.55 | 1.00 |  |  |  |  |  | Dominant | 2.96 | 0.67 |  |  |  |
|  | Submissive ^b^ | 3.95 | 0.88 |  |  |  |  |  | Submissive | 2.78 | 0.66 |  |  |  |
|  | Switching ^b^ | 3.95 | 0.72 |  |  |  |  |  | Switching | 2.87 | 0.69 |  |  |  |
| **CHA** |  |  |  | 5.67 | **.004** | .05 |  | **THR** |  |  |  | 1.79 | .170 | .02 |
|  | Dominant ^c^ | 3.12 | 0.82 |  |  |  |  |  | Dominant | 2.33 | 0.55 |  |  |  |
|  | Submissive ^d^ | 3.65 | 0.85 |  |  |  |  |  | Submissive | 2.37 | 0.65 |  |  |  |
|  | Switching ^d^ | 3.64 | 0.78 |  |  |  |  |  | Switching | 2.53 | 0.60 |  |  |  |
| **WAR** |  |  |  | 0.79 | .457 | .01 |  | **HEL** |  |  |  | 0.12 | .887 | < .01 |
|  | Dominant | 4.16 | 0.52 |  |  |  |  |  | Dominant | 2.41 | 0.68 |  |  |  |
|  | Submissive | 4.27 | 0.51 |  |  |  |  |  | Submissive | 2.34 | 0.66 |  |  |  |
|  | Switching | 4.28 | 0.43 |  |  |  |  |  | Switching | 2.36 | 0.57 |  |  |  |
| **ACS** |  |  |  | 0.05 | .956 | < .01 |  | **PUN** |  |  |  | 0.24 | .784 | < .01 |
|  | Dominant | 2.93 | 0.77 |  |  |  |  |  | Dominant | 1.86 | 0.71 |  |  |  |
|  | Submissive | 2.95 | 0.73 |  |  |  |  |  | Submissive | 1.90 | 0.82 |  |  |  |
|  | Switching | 2.97 | 0.74 |  |  |  |  |  | Switching | 1.97 | 0.80 |  |  |  |
| **SEC** |  |  |  | 1.15 | .319 | .01 |  | **OBS** |  |  |  | 1.49 | .23 | .01 |
|  | Dominant | 2.54 | 0.79 |  |  |  |  |  | Dominant | 2.92 | 0.80 |  |  |  |
|  | Submissive | 2.72 | 0.69 |  |  |  |  |  | Submissive | 2.68 | 1.02 |  |  |  |
|  | Switching | 2.76 | 0.62 |  |  |  |  |  | Switching | 2.90 | 0.91 |  |  |  |

*Note*: Dominant: *N* = 32. Submissive: *N* = 113. Switching: *N* = 69. *df* = 2, 211. ^a-d^ Groups which do not share the same letter differ significantly (Tukey post-hoc tests). Significant *p*-values are printed in bold.

**Table F**. Hierarchical logistic regression predicting SM sexual preference (submissive and switching roles only).

| Model | Predictor | % correctly classified | Nagelkerke  R^2^ | β | *SE* | *OR* | *Wald* | *p* |
| --- | --- | --- | --- | --- | --- | --- | --- | --- |
| **0** | Constant | **54.0** | - |  |  |  |  |  |
|  |  |  |  | -0.16 | 0.10 | 0.85 | 2.58 | .108 |
| **1** |  | **59.8** | **.05** |  |  |  |  |  |
|  | Constant |  |  | < -0.16 | 0.10 | 0.85 | 2.39 | .122 |
|  | CP status |  |  | 0.37 | 0.10 | 1.44 | 12.67 | **< .001** |
|  | PSQ total |  |  | -0.11 | 0.10 | 0.90 | 1.10 | .259 |
| **2** |  | **66.2** | **.23** |  |  |  |  |  |
|  | Constant |  |  | -0.15 | 0.11 | 0.86 | 1.82 | .177 |
|  | CP status |  |  | 0.52 | 0.12 | 1.69 | 20.47 | **< .001** |
|  | PSQ total |  |  | -0.04 | 0.11 | 0.96 | 0.12 | .961 |
|  | SSS-V total |  |  | 0.92 | 0.13 | 2.52 | 49.46 | **< .001** |
| **3** |  | **76.3** | **.44** |  |  |  |  |  |
|  | Constant |  |  | -0.13 | 0.13 | 0.81 | 2.92 | .806 |
|  | CP status |  |  | 0.56 | 0.13 | 1.75 | 18.49 | **< .001** |
|  | PSQ total |  |  | 0.02 | 0.13 | 1.02 | 0.01 | .909 |
|  | SSS-V total |  |  | 0.66 | 0.15 | 1.94 | 20.60 | **< .001** |
|  | GATPI CHA |  |  | 1.21 | 0.17 | 3.35 | 53.42 | **< .001** |
|  | GATPI ACS |  |  | 0.06 | 0.14 | 1.06 | 0.18 | .806 |

*Note.* *N* = 396. OR = Odds ratio. CP = Chronic pain. PSQ = Pain Sensitivity Questionnaire. SSS-V = Sensation Seeking Scale Form V. GATPI = General Attitudes Towards Pain Inventory. FAS-PLE = Fascination-Pleasure. ACS = Acceptance & Stoicism. TRA = Tragedy. SM: 0 = no, 1 = yes. CP status: 0 = no, 1 = yes.


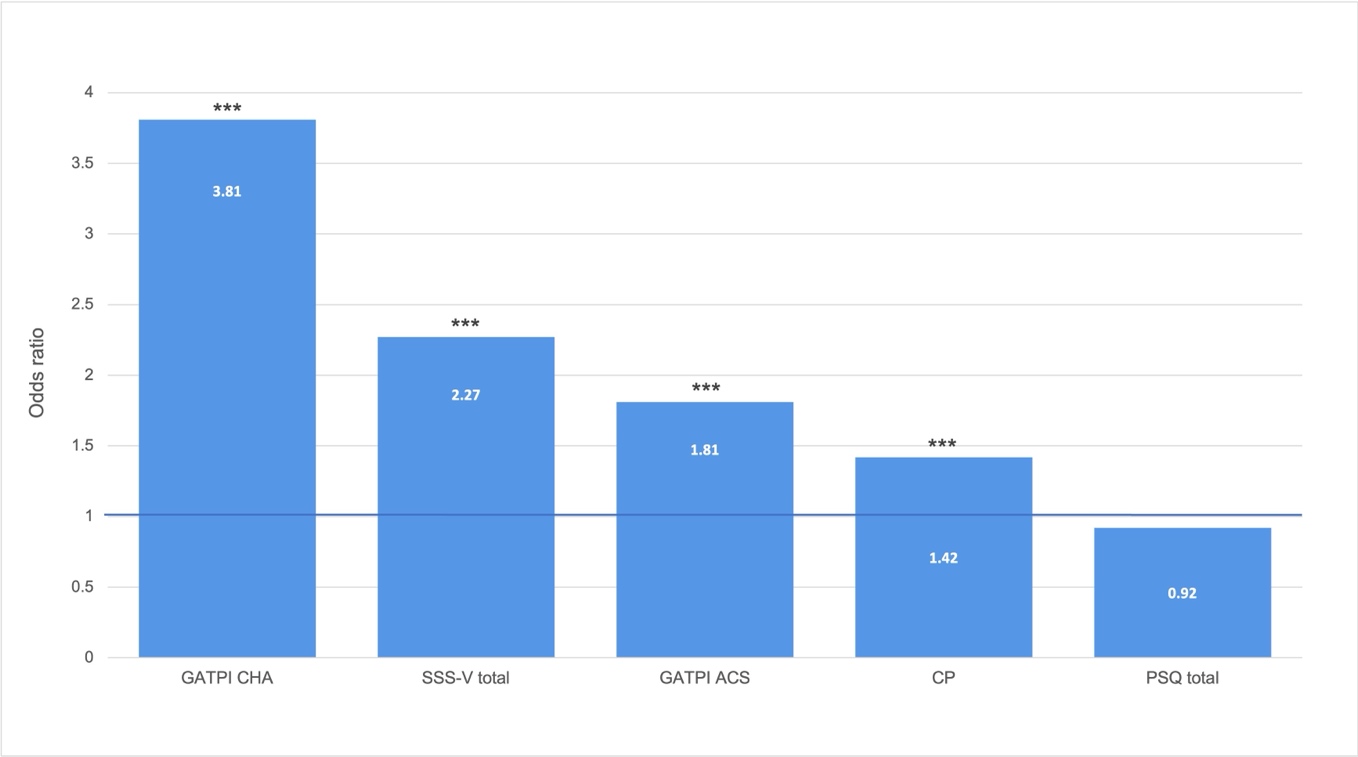


**Figure B**. Odds ratios of variables predicting sadomasochistic sexual preference (submissive and switching roles only, odds ratios are presented in descending order). GATPI = General Attitudes Towards Pain Inventory. CHA = Challenge. SSS-V = Sensation Seeking Scale Form V. ACS = Acceptance & Stoicism. CP = Chronic pain. PSQ = Pain Sensitivity Questionnaire. Significance of predictors: *** *p* < .001
